# Manure and mosquitoes: life history traits of two malaria vector species enhanced by larval exposure to cow dung, whilst chicken dung has a strong negative effect

**DOI:** 10.1186/s13071-022-05601-3

**Published:** 2022-12-16

**Authors:** Harrison Hardy, Richard Hopkins, Ladslaus Mnyone, Frances M. Hawkes

**Affiliations:** 1 Natural Resources Institute, University of Greenwich, London, UK; 2 Institute of Pest Management, Sokoine University of Agriculture, Morogoro, Tanzania; 3 Department of Science, Technology and Innovation, Ministry of Education, Science and Technology, Dar Es Salaam, Tanzania

**Keywords:** Organic fertilisers, *Anopheles arabiensis*, *Anopheles gambiae* sensu stricto, Rice cultivation, Malaria vectors, Mosquito larvae, Life history traits

## Abstract

**Background:**

Malaria vectors have a strong ecological association with rice agroecosystems, which can provide abundant aquatic habitats for larval development. Climate-adapted rice cultivation practices, such as the System of Rice Intensification (SRI), are gaining popularity in malaria-endemic countries seeking to expand rice production; however, the potential impact of these practices on vector populations has not been well characterised. In particular, SRI encourages the use of organic fertilisers (OFs), such as animal manures, as low-cost and environmentally friendly alternatives to industrially produced inorganic fertilisers. We therefore set out to understand the effects of two common manure-based OFs on the life history traits of two major African malaria vectors, *Anopheles arabiensis* and *Anopheles gambiae* sensu stricto (s.s.).

**Methods:**

Larvae of *An. arabiensis* and *An. gambiae* s.s. were reared from first instar to emergence in water containing either cow or chicken dung at one of four concentrations (0.25, 0.5, 0.75, and 1.0 g/100 ml), or in a clean water control. Their life history traits were recorded, including survival, development rate, adult production, and adult wing length.

**Results:**

Exposure to cow dung significantly increased the development rate of *An. gambiae* s.s. independent of concentration, but did not affect the overall survival and adult production of either species. Chicken dung, however, significantly reduced survival and adult production in both species, with a greater effect as concentration increased. Interestingly, *An. arabiensis* exhibited a relative tolerance to the lowest chicken dung concentration, in that survival was unaffected and adult production was not reduced to the same extent as in *An. gambiae* s.s. The effects of chicken dung on development rate were less clear in both species owing to high larval mortality overall, though there was some indication that it may reduce development rate. Adult wing lengths in males and females increased with higher concentrations of both cow and chicken dung.

**Conclusions:**

Our findings suggest that manure-based OFs significantly alter the life history traits of *An. gambiae* s.s. and *An. arabiensis.* In both species, exposure to cow dung may improve fitness, whereas exposure to chicken dung may reduce it. These findings have implications for understanding vector population dynamics in rice agroecosystems and may inform the use of OFs in SRI, and rice agriculture more widely, to avoid their adverse effects in enhancing vector fitness.

**Graphical Abstract:**

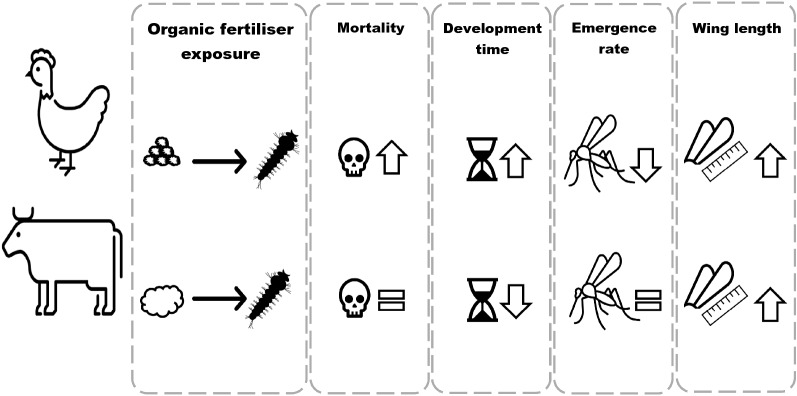

## Background

Rice cultivation and malaria are inextricably linked, as standing water within the agroecosystem provides conducive breeding habitats for malaria vectors [1–3]. It has long been recognised that rice farming can lead to increased malaria vector populations, although it has been suggested that the associated economic benefits of rice cultivation can lead to improved access to preventative control measures and healthcare, which offset any possible increase in malaria transmission [4]. However, more recent studies have revealed that, over the past two decades, this has not been the case and that communities associated with rice cultivation experience a greater malaria burden, although the exact mechanisms behind this change have yet to be fully elucidated [5].

As part of their ongoing effort to increase rice production [6, 7], several malaria-endemic countries in sub-Saharan Africa are adopting alternative rice cultivation practices, such as the System of Rice Intensification (SRI) [8]. Whilst SRI is relatively more laborious, it promises greater yields with reduced agricultural inputs, although this claim is debated among agronomists [9–11]. With the continuing threat of climate change, the application of organic fertilisers (OFs) within rice cultivation fields, replacing or supplementary to inorganic fertilisers, has been suggested to mitigate greenhouse gas emissions [12]. Within SRI, OFs are promoted as low-cost fertiliser formats due to their wide availability and lower cost compared to inorganic fertilisers [13, 14], although their composition is poorly characterised [15]. Typically, they comprise readily available organic material, such as vegetative matter or livestock dung [13, 16]. Cow and chicken dung are common OFs in traditional rice cultivation [17, 18], but within SRI, cow dung and rice straw are the most common [19]. Moreover, cow and chicken dung are among the most popular OFs used in urban agricultural settings for rice cultivation [20].

The presence of organic matter in larval breeding water is known to have important impacts on *Anopheles* mosquitoes, such as larval to adult development rate, which may be hastened or slowed [21, 22], larval survivorship, which may be enhanced or diminished [23, 24], and adult fitness, which may be improved or reduced [21, 25–27], all of which are highly dependent on the type and amount of organic matter present, and associated microbial communities. These are important factors in the vectorial capacity of a given population, and hence, malaria transmission intensity [28]. Moreover, organic matter content in larval habitats has been found to influence the susceptibility of *An. gambiae* sensu lato (s.l.) to *Plasmodium* infection and, consequently, malaria transmission [25]. Despite this, little attention has been given to the study of how organic material in the form of manure-based fertilisers may impact anopheline mosquitoes.

*Anopheles arabiensis*’ preference for cattle hosts and the tendency of both *An. arabiensis* and *An. gambiae* s.s. to colonise hoofprint puddles [29–31] may increase their likelihood of encountering cattle excretions. In field studies, eutrophication due to the presence of cow dung has been suggested to increase mosquito oviposition and development rates in *Anopheles* species*.* [1, 32]. Whilst few controlled laboratory studies have been carried out to date, larval exposure to the cow dung derivative Fertilis was found to significantly increase development rate, adult longevity, adult size, and insecticide tolerance in *An. gambiae* s.l. [21]. However, another study demonstrated that cow dung causes significant mortality in anopheline larvae [33]. The relationship between cow dung and *An. gambiae* s.l., therefore, seems to be complex and at present unclear. There is considerably less data on the potential impact of chicken dung on anopheline fitness, although a small body of research has shown both stimulatory and repellent oviposition effects in some *Culex* species [34, 35]. The effect of chicken dung on mosquitoes, in general, remains unclear, and in *Anopheles* species, understudied.

Considering this, it is important to explore the impact of cow and chicken dung as OFs on the life history traits of *Anopheles* mosquitoes. In comparison to research on inorganic fertilisers, that on OFs is scant and its results contradictory. As the impacts of climate change continue to affect agriculture, the adoption of climate-adapted practices such as SRI, or an increased reliance on more ecologically sustainable fertilisers, such as waste materials like animal dung, is increasingly likely. It is, therefore, important to understand how OFs may impact malaria vector species and the transmission of malaria. In this study, larval exposure of *An. gambiae* s.s. and *An. arabiensis*, which are major malaria vectors of Africa, to cow and chicken dung was studied to determine their effects on the species’ life history traits, specifically larval survival and development rate, and adult production and wing length. The results are discussed within the context of how malaria transmission may be influenced by these effects on the vectors.

## Methods

### Mosquitoes

Mosquitoes were maintained in a climate-controlled insectary at 27.5 ± 2 °C and 50 ± 10% relative humidity, with a 12:12 light–dark photoperiod. *Anopheles gambiae* s.s. were obtained from an established colony originally sourced from Burkina Faso. *Anopheles arabiensis*, Dongola strain, were obtained from an established colony at the International Atomic Energy Association (Austria). Larvae were reared in isotonic water (distilled water with 0.1% aquarium salts) and fed Tetramin fish flakes ad libitum. Adults were maintained on 10% sucrose solution, feeding ad libitum, and females were offered a human blood meal for egg production at 7–10 days old.

### Preparation of OF material

Cow dung was sourced from an organic farm in Kent, UK, from Irish Holstein–Friesian cows (*Bos taurus*). No ivermectin had been administered to the cows in over 6 months, nor any other medications. Their diet comprised organic non-pesticide-treated feed only, consisting of grass, bean sprouts, and barley in summer, and silage and protein powder in winter. Chicken dung was sourced from domestic chickens (*Gallus gallus* var. *domesticus*) kept by a private hobbyist in Kent. The chickens had not been treated with any medications, except for one dose of a coccidiostat as chicks, 2 years prior to dung collection. Their diet comprised organic non-pesticide-treated feed, chicken pellets, corn, and vegetable scraps. Prior to experimentation, the cow and chicken dung was dried for 24 h at 60 °C, then coarsely ground using a 150-Watt spice grinder (Wahl). It was then stored at 4 °C in airtight jars, with a small amount of silica gel, which was kept separate from the dung.

### Experimental pot design

**Fig. 1:**
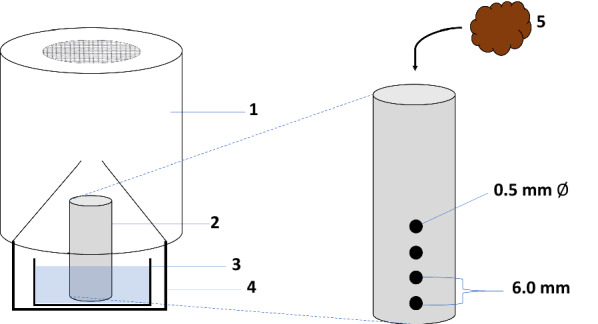
Schematic diagram of the experimental pot assembly used in the bioassays. Left: Top half of a BioQuip breeder pot (*1*), 5-ml specimen tube (*2*), 75-ml plastic dish (*3*), 200-ml plastic pot (*4*). Right: Expanded schematic diagram of the 5-ml specimen tube, showing drilled hole dimensions; organic fertiliser material (*5*)

Initial experiments showed that placing dung directly into rearing water significantly obscured our view of the larvae, making counting first and second instars difficult and unreliable. Therefore, a holder made from an adapted specimen tube was developed (Fig. 1). Briefly, sixteen 1.5-mm-diameter holes were drilled into a 5-ml specimen tube (54 mm × 15 mm diameter). The holes were arranged in four equidistant columns of four holes; the holes in each column were 6 mm apart, with the first hole 6 mm from the bottom of the tube. The tube was then filled with the required weight of dung and placed into a 75-ml (33 mm × 60 mm diameter) plastic dish with 50 ml of isotonic water. The dish was then placed in a 200-ml plastic pot (47 mm × 95 mm diameter), and the top half of a BioQuip breeder pot (195 mm × 92 mm diameter) was placed over it to collect emerging adults. Dung was added 24 h before the experiments commenced to allow it to fully hydrate.

### Bioassays

Laboratory bioassays were conducted to measure the survival of immatures (larvae and pupae), development rate, adult production, and adult body size in response to cow and chicken dung exposure. A separate bioassay was conducted for each species and dung type combination, following a randomised block design. Each bioassay consisted of five treatment groups: a control group, which received no dung infusion, and four experimental treatment groups representing exposure to OF concentrations of 0.25, 0.5, 0.75, and 1.0 g/100 ml. Each treatment was replicated ten times in each bioassay. Ten L1 larvae, less than 24 h old, were placed into each experimental pot at the start of each bioassay. Larvae were fed Tetramin fish flakes every day at a rate of 0.3 mg/larva [36]. Daily counts were made of the number of live and dead larvae and pupae, and emerged adults. Each pot was assigned a number from 1–50 and daily counts were performed in random order each day. Any dead individuals were immediately removed. Emerged adults were preserved for wing length measurement.

### Determination of adult body size

Wing length was measured, as a proxy for adult size, from the alular notch to the intersection of the third radial vein and the costa at the apex, excluding the wing fringe scales [37, 38]. As left and right wing lengths are highly correlated in *An. gambiae* s.l. [38], only one wing per individual was measured. All measurements were conducted at ×40 magnification using a GXM ultraZOOM-2 Stereo Microscope Series 8X-50X Trinocular fitted with a GXCAM-U3 C mount digital camera (GT Vision). Images were captured and wing length measurements were made using the GX Capture-T software (GT Vision). For each treatment group, ten males and ten females were randomly selected for wing length measurement, except for the *An. arabiensis* exposed to 0.5 g/100 ml of chicken dung (six females and five males) and the *An. gambiae* s.s. exposed to 0.25 g/100 ml chicken dung (10 females and 15 males), where all were measured due to low adult production.

### Statistical methods

All statistical analyses were performed in R (version 3.6.2, RStudio version 1.2.5033) [39]. Kaplan-Meir survival estimator models were used to examine the effects of dung on survival. Log rank tests were performed to test the independence of survival curves using Holm’s adjustment for multiple comparisons [40]. The restricted mean survival time (hereafter referred to as mean survival time), up to day 10, was used as the descriptive statistic to summarise the differences in survival between the dung treatment groups [41]. Quasi-binomial models were fitted to analyse the effects of dung on adult emergence rate [42]. Larval to adult development rates were analysed using Friedman’s analysis of variance followed by pairwise Wilcoxon signed rank tests for post hoc analysis, with Holm’s adjustment for multiple comparisons [43]. Differences in wing length were analysed using an analysis of covariance, with sex as the covariate. Post hoc testing was performed using Dunnett’s test [44]. For both development and wing length analysis, the observations for *An. arabiensis* treated at 0.75 and 1.0 g/100 ml of chicken dung were excluded from the analyses as the former concentration led to only a single male being produced, and in the latter no adults were produced. For *An. gambiae* s.s., observations for chicken dung treatment concentrations from 0.5 g/100 ml and above were not included as no adults were produced.

## Results

### Effect on immature mosquito survival

**Fig. 2:**
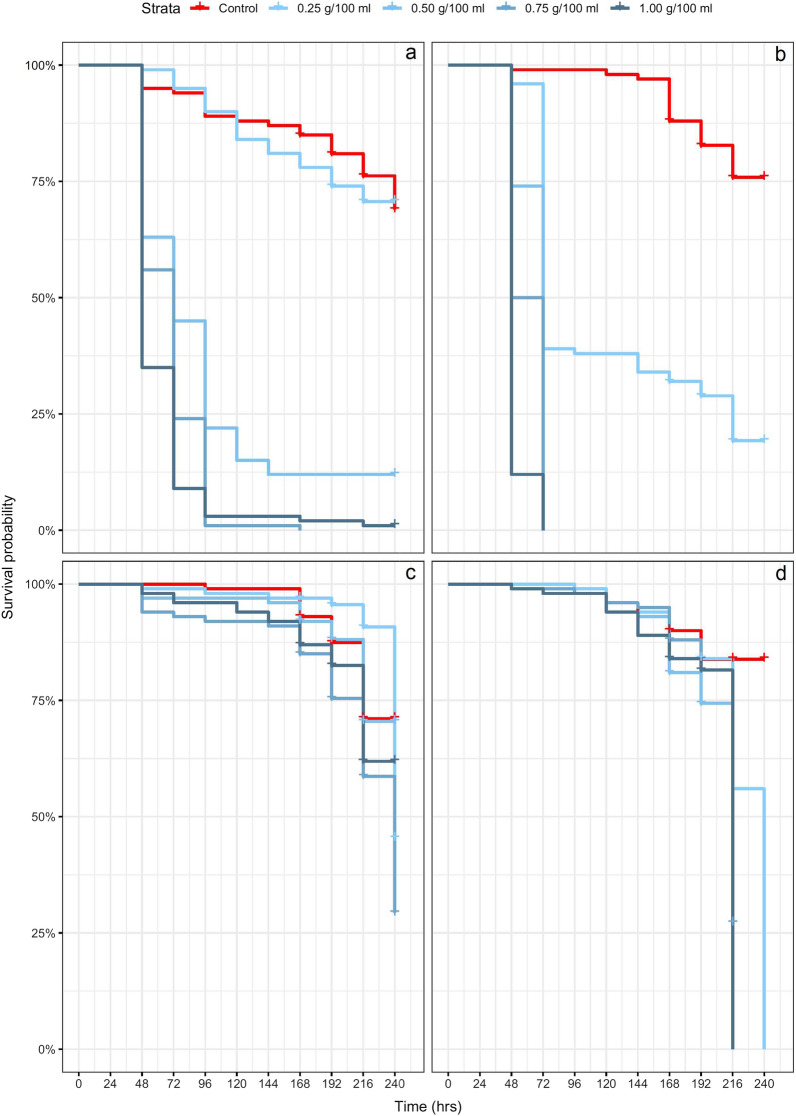
Survival probabilities over time of larval *Anopheles arabiensis* (**a**, **c**) and *Anopheles gambiae* sensu stricto (s.s.) (**b**, **d**) exposed to different concentrations of chicken dung (**a**, **b**) and cow dung (**c**, **d**), based on a Kaplan-Meir survival estimator model. Crosses indicate censoring events (emergence to adult)

Chicken dung had significant effects on the survival of both *An. arabiensis* and *An. gambiae* s.s., though this was concentration dependant and varied by species. In *An. arabiensis*, exposure to 0.25 g/100 ml (*χ*^2^ = 0.5, *df* = 1, *P* = 1) did not lead to any significant difference in survival; however, exposure to chicken dung at 0.5 g/100 ml (*χ*^2^ = 39.2, *df* = 1, *P* ≤ 0.001), 0.75 (*χ*^2^ = 79.2, *df* = 1, *P* ≤ 0.01), and 1.0 g/100 ml (*χ*^2^ = 78.5, *df* = 1, *P* ≤ 0.001) resulted in significantly lower survival compared to the control (Fig. 2a). The mean survival time was 214.83 (± 5.47) h for the control group and 209.20 (± 5.47) h for the group exposed to 0.25 g/100 ml. For the groups exposed to 0.5, 0.75 and 1.0 g/100 ml of chicken dung, mean survival times were 94.32 (± 5.96), 67.92 (± 2.15), and 61.92 (± 2.98) h, respectively.

Likewise, exposure to chicken dung resulted in significantly lower survival in *An. gambiae* s.s. at 0.25 g/100 ml (*χ*^2^ = 23.9, *df* = 1, *P* ≤ 0.001), 0.5 g/100 ml (*χ*^2^ = 88.1, *df* = 1, *P* ≤ 0.001), 0.75 g/100 ml (*χ*^2^ = 93.3, *df* = 1, *P* ≤ 0.01), and 1.0 g/100 ml (*χ*^2^ = 107, *df* = 1, *P* ≤ 0.001) (Fig. 2b). The mean survival time for the control was 225.27 (± 3.51) h, and for the chicken dung treatment groups 126.04 (± 7.39), 65.76 (± 1.05), 60.0 (± 1.20), and 50.88 (± 0.78) h, respectively, for 0.25, 0.5, 0.75 and 1.0 g/100 ml. These data suggest that exposure to chicken dung reduced survival in the immature aquatic stages of both *An. arabiensis* and *An. gambiae* s.s.; however, the former species may possess a relative tolerance for lower chicken dung concentrations.

Larval exposure to cow dung did not lead to any significant difference in survival in either *An. arabiensis* or *An. gambiae* s.s. at any of the concentrations tested (Fig. 2c, d).

### Effect on immature mosquito development

**Fig. 3:**
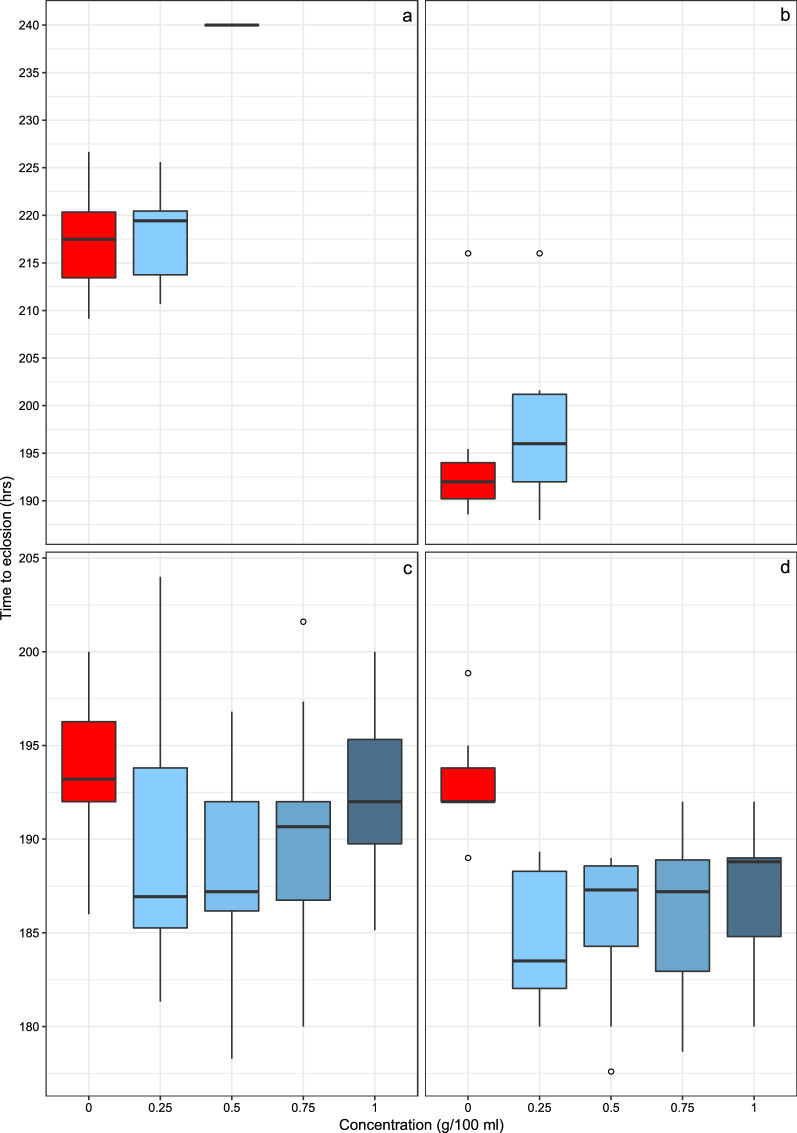
Development rate, as the time to eclosion, of *Anopheles arabiensis* (**a**, **c**) and *Anopheles gambiae* s.s. (**b**, **d**) when exposed to different concentrations of chicken dung (**a**, **b**) and cow dung (**c**, **d**). Boxes show the interquartile range of development rates, the median is shown as a solid black line, and outliers as white circles

For the *An. arabiensis* immatures exposed to chicken dung, a significant difference in median development rate between the treatment groups and the control was observed (*χ*^2^ = 11.143, *df* = 2, *P* ≤ 0.01) (Fig. 3a). Treatment with 0.25 g/100 ml resulted in a median development rate of 219.4 h, which was not statistically different from the 217.5 h observed in the control (*Z* = −1.418, *P* = 0.922). However, at 240 h, larvae treated at 0.5 g/100 ml had developed significantly more slowly (*Z* = −1.701, *P* ≤ 0.05). This suggests that, in *An. arabiensis*, exposure to intermediate concentrations of chicken dung caused eclosion to occur almost a full day later than lower concentrations or no chicken dung at all. In *An. gambiae* s.s., only the control group and those treated at 0.25 g/100 ml of chicken dung produced adults; however, no significant difference in development rate was observed between them (Fig. 3b), with median development rates of 196 and 192 h, respectively.

Across all treatment concentrations, larval *An. arabiensis* exposed to cow dung showed no significant difference in their development rate, measured as median time to eclosion, relative to the control. In contrast, *An. gambiae* s.s. exhibited a significant difference in development rate between the treatment groups (*χ*^2^ = 16.944, *df* = 4, *P* ≤ 0.01) (Fig. 3d). All dung treatment concentrations led to a significantly shorter time to eclosion compared to the control, which had a development rate of 192.0 h. The shortest time to eclosion, 183.5 h, was observed in larvae exposed to 0.25 g/100 ml (*Z* = −2.154, *P* = 0.016), whilst those exposed to 1.0 g/100 ml (*Z* = −2.093, *P* = 0.018) had the slowest development time of the treatment groups, at 188.8 h. Exposure to 0.5 g/100 ml (*Z* = −2.111, *P* = 0.017) and 0.75 g/100 ml (*Z* = −0.2093, *P* = 0.018) resulted in development rates of 187.3 and 187.2 h, respectively. These data indicate that exposure to cow dung at the concentrations tested significantly reduces the time to eclosion in *An. gambiae* s.s., although the difference between this for each treatment group and the control was less than 10 h.

### Effect on adult production

**Fig. 4:**
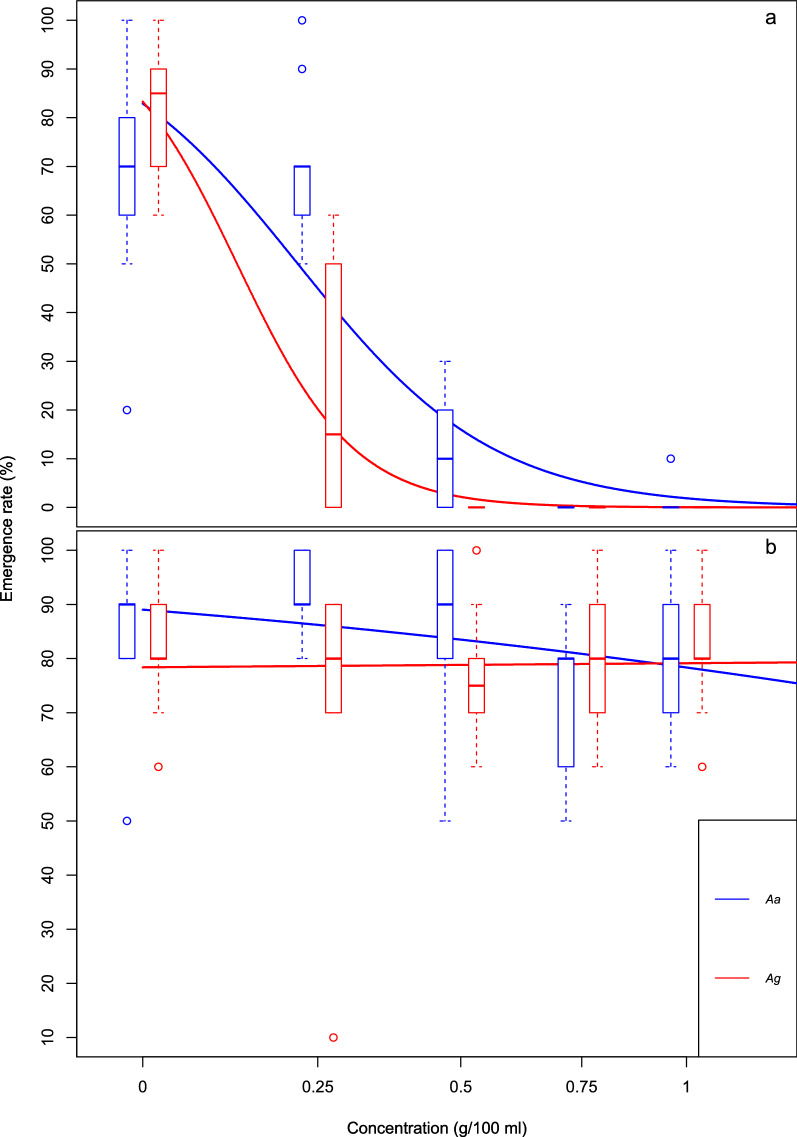
Emergence rate in *Anopheles arabiensis* (*Aa*) and *Anopheles gambiae* s.s. (*Ag*) when exposed to chicken dung (**a**) and cow dung (**b**) at different concentrations. Boxplots show percentage emergence rates, with the median displayed as solid coloured lines and outliers as coloured circles. Curves show fitted logit regression lines from the quasi-binomial generalised linear model

Chicken dung had a highly significant effect on adult emergence rate [*F*_(1,98)_ = 314.8624, *P* ≤ 0.001], where dung concentration and emergence rate were inversely related (Fig. 4a). In addition, the effect of concentration on emergence rate differed by species [*F*_(1,96)_ = 9.4885, *P* ≤ 0.01]. Overall, with each 0.25 g/100 ml increase in chicken dung concentration, the emergence rate of *An. arabiensis* was higher than that of *An. gambiae* s.s., with the greatest difference between 0.25 and 0.5 g/100 ml. These data suggest that an increase in chicken dung concentration reduces the emergence rate in both species, but that this reduction is greater in *An. gambiae* s.s. than in *An. arabiensis.* In contrast, cow dung concentration did not significantly affect adult emergence rate in either species, nor was there a significant difference in this between the species (Fig. 4b).

### Effect on adult body size

**Fig. 5:**
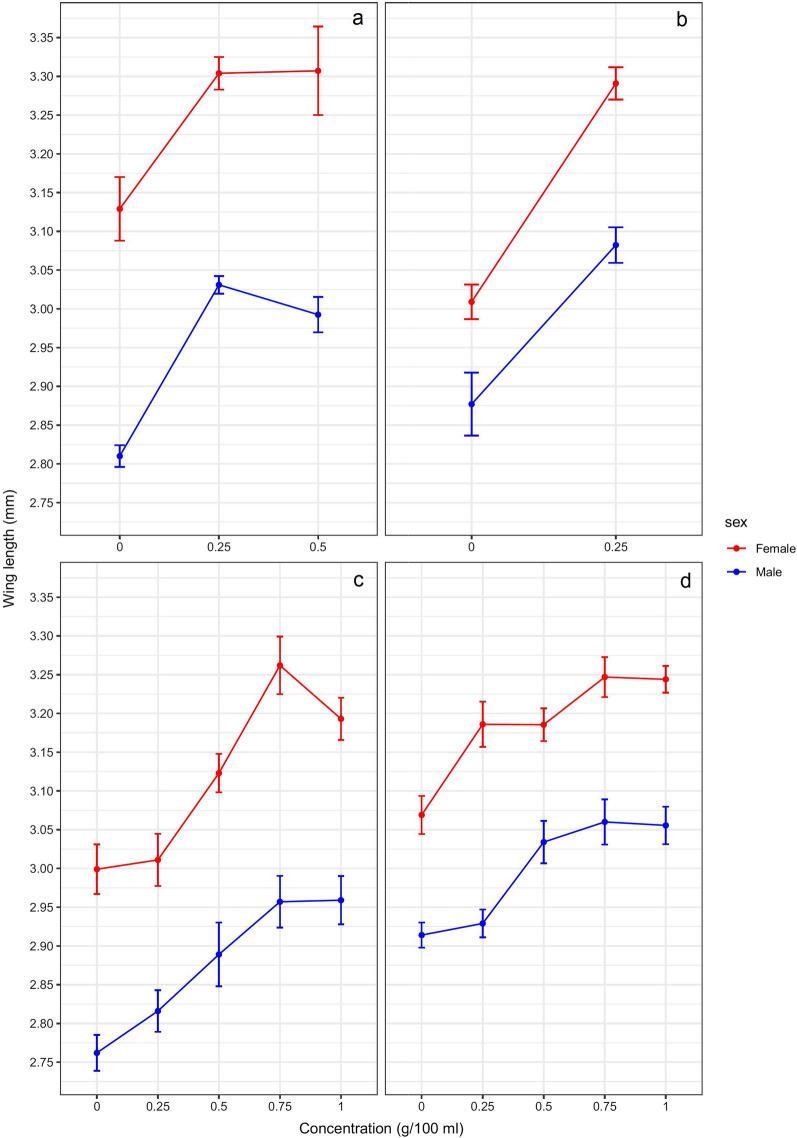
Mean adult female and male mosquito wing length (± SE) of *Anopheles arabiensis* (**a**, **c**) and *Anopheles gambiae* s.s. (**b**, **d**) after larval exposure to different concentrations of chicken dung (**a**, **b**) and cow dung (**c**, **d**)

In *An. arabiensis,* chicken dung concentration [*F*_(2,45)_ = 32.496, *P* ≤ 0.01] and sex [*F*_(1,45)_ = 139.522, *P* ≤ 0.01] had significant effects on mean wing length, but no significant interaction between dung concentration and sex was found [*F*_(2,45)_ = 0.371, *P* = 0.692]. In both males and females, mean wing length was significantly increased, compared to the control, across all concentrations (all, *P* ≤ 0.01) (Fig. 5a). Chicken dung concentration [*F*_(1,40)_ = 74.688, *P* ≤ 0.01] and sex [*F*_(1,40)_ = 40.995, *P* ≤ 0.01] also had significant effects on mean wing length in *An. gambiae* s.s., again with no significant interaction between the two observed [*F*_(1,40)_ = 1.979, *P* = 0.167]. Post hoc comparisons showed that exposure to 0.25 g/100 ml chicken dung led to significantly larger mean wing length in both males and females (all, *P* ≤ 0.001) (Fig. 5b).

In the *An. arabiensis* exposed to cow dung, there were significant effects of dung concentration [*F*_(4,90)_ = 19.877, *P* ≤ 0.01] and sex [*F*_(1,90)_ = 146.054, *P* ≤ 0.01] on mean wing length. However, no significant interaction between concentration and sex was observed [*F*_(4,90)_ = 0.795, *P* = 0.531]. For both females and males, exposure to all cow dung concentrations led to increased mean wing length compared to the control; however, this increase was only statistically significant in those exposed to 0.5 g/100 ml and above (all, *P* ≤ 0.05), in both males and females (Fig. 5c). Likewise, cow dung concentration [*F*_(4,90)_ = 17.147, *P* ≤ 0.01] and sex [*F*_(1,90)_ = 156.096, *P* ≤ 0.01] also had significant effects on mean wing length in *An. gambiae* s.s., but again, no significant interaction between concentration and sex was found [*F*_(4,90)_ = 1.612, *P* = 0.178]. Mean wing length increased compared to the control in females and males when they were exposed to all cow dung concentrations. In females, all concentrations lead to significantly larger wing lengths than in the control (all, *P* ≤ 0.05), whilst in males wing lengths were significantly larger in those exposed to 0.5 g/100 ml and higher concentrations (all, *P* ≤ 0.05) (Fig. 5d). These data suggest that wing length, and therefore adult body size, increase in both species when they are exposed to cow and chicken dung as larvae.

## Discussion

Cow and chicken dung differentially affected the survival, adult production and development rate of the mosquitoes exposed to them in their aquatic larval stages. In general, cow dung had a net positive effect on these life history traits, whilst chicken dung had a net negative effect. However, one consistent effect of the two types of OF was that they increased adult wing length in both the species tested, where larvae exposed to higher dung concentrations developed into larger adults. Nevertheless, other important differences in life history traits were observed between *An. arabiensis* and *An. gambiae* s.s. Larval exposure to cow dung over all treatment concentrations did not impact survival in either *An. gambiae* s.s. or *An. arabiensis*. This result corroborates and adds to findings of a laboratory study which showed that Fertilis, a cow dung derivative, had no effect on *An. gambiae* s.l. mortality [21].

Much of the research into cow dung’s effects on *Anopheles* mosquitoes has been semi-field based, but this represents a small body of work. Observations in Kenya showed no impact of cow dung exposure on the survival of *An. gambiae* s.s. larvae [45], and the addition of cow dung to outdoor mesocosms in Botswana was found to increase anopheline larval abundance, with increasing effects at higher dung concentrations [32]. Similar observations were found in The Gambia, where cow dung addition to potential breeding environments resulted in increased abundances of anopheline larvae, including *An. arabiensis* and *An. gambiae* s.s. larvae [1]. However, the results of similar studies are contradictory, as the presence of cow dung was found to be associated with increased larval mortality in *Anopheles* species [33] and reduced abundances of *Anopheles gambiae* s.l. and *Anopheles funestus *s.l. larvae [23]. These disparities are likely due to methodological differences and because studies which observed no effect on survival or abundance of Anopheles species used lower cow dung dosages than those that reported increased mortality or reduced abundances. In addition, the dietary composition of the feed given to the cows from which the dung was used in each respective study, and the age of the dung or infusions also likely contributed to the observed discrepancies. Moreover, as a laboratory-controlled experiment, the findings of the present study are based only on the direct effects of dung exposure, whereas in field-based studies many more ecological factors, such as predation, competition, and environmental changes, will significantly influence observed outcomes.

In the present study, cow dung exposure led to faster development in both *An. arabiensis* and *An. gambiae* s.s., though this was only statistically significant in the latter species and represented a relatively small difference from the control. Nevertheless, these results correspond with those of Jeanrenaud et al. [21], who found that cow dung, used at similar dosages to this study, increased development rates in *Anopheles gambiae* s.l., although these increases were similarly small. While the results of a semi-field trial suggested that cow dung may only increase the development rate of *Anopheles* species when nutritional resources are limited [45], in the present study the availability of food was strictly controlled according to larval density, yet this effect was still observed. In addition to nutrient availability, the larval development rate of *Anopheles* mosquitoes is influenced by the average ambient temperature [46–51]. As temperatures were controlled in this study, it is likely that faster development rates arose from cow dung acting as an additional food source, as alluded to by other authors [21, 32]. However, this effect did not increase with higher dung concentrations, suggesting that even the lowest dose in this study may represent an upper limit to which cow dung exposure benefits *Anopheles* mosquito development rate. Alternatively, a more complex, hormetic mechanism may underlie these observations, whereby increasing cow dung concentrations confer a disadvantage that negates any possible developmental benefits [52]. It is unclear how dosages higher than those used in this study, and possible under field conditions, would impact the development rate of larvae, and thus warrant further study.

The use of chicken manure as an OF in aquatic agroecosystems is well established, as it has the highest nitrogen, phosphorous, and potassium content of most commonly available animal manures whilst also being highly soluble [53]. It has been found to promote the growth of algae, phytoplankton, and aquatic invertebrates, including Culicidae [54]. Notably, avian species excrete nitrogenous waste as uric acid along with their faeces, whilst mammalian species excrete the majority of their nitrogenous waste via urination. However, to date, to the best of our knowledge, no published research has examined the effects of chicken dung on the life history traits of *Anopheles* mosquitoes. The present study has demonstrated that chicken dung exposure is non-conducive to the development of immature *An. gambiae* s.s. and *An. arabiensis.* Exposure to chicken dung in the immature stages led to reduced survival and adult production, where both decreased with increasing dung concentration. Additionally, exposure to chicken dung at the different concentrations, apart from the very lowest, reduced the development rate of *An. arabiensis*, although it was unclear whether *An. gambiae* s.s. was similarly affected due to its high mortality under the test conditions. However, failure to reach adulthood can be viewed as a significant, if not total, disruption of development, rather than as delayed development.

At the very lowest chicken dung concentration, the development rate of both species was not impacted. However, at the higher concentrations, very few larvae survived, and those that did survive to the adult stage took significantly longer to reach eclosion, although this was only demonstrated in *An. arabiensis*. Given, as discussed above, that the developing larvae may have consumed the OFs, an increased rate of development could have been expected as a consequence of nutritional reserves accumulating faster; however, this was not observed. Most of the nitrogen content of chicken dung is in the form of urea and uric acid [55], and the uric acid is readily converted to urea by endogenous bacteria [56]. Exposure to urea significantly reduces the development rate of *An. arabiensis* larvae [57], which may explain its reduced development rate in the present study. In comparison to cow dung, chicken dung contains twice the amount of soluble nitrogen, 26 times the amount of phosphorous, and three times the amount of potassium [54]. However, the exact effect that each nutritive element may have on developing mosquitoes remains unclear [57]. Although the present study demonstrated that chicken dung clearly did not benefit mosquito fitness and was detrimental to their survival and development, if it did contribute to the nutritive content of the water, be that directly for the developing larvae or indirectly via aquatic microbiota that the larvae may have fed on, any benefit was far outweighed by other factors that reduced fitness. Quantitating the nutritive content of dung-infused water may be of significant interest for future attempts to explain the observations made in this study.

Whilst it is presently unclear what the causative agent of chicken dung’s toxicity is, one explanation for its toxicity may be the presence of pathogenic bacteria. Chicken dung is associated with double the total bacterial count of cow dung, and mainly contains *Bacillus* species [53]. There is a large diversity of *Bacillus* species that produce endotoxins with mosquitocidal properties, many of which are endogenous to larval habitats and reduce mosquito survival [58–62]. Moreover, *Bacillus thuringiensis* has been isolated from chicken dung samples [63], and chickens have been identified as both host and source of the bacterium in their faeces [64]. As *An. gambiae* s.l. larvae consume OFs [21, 32], should an abundance of endotoxin-producing bacteria have been present in the chicken dung, the mosquito larvae may have consumed them, resulting in the observed lethal effects; however, it was not possible to measure the bacterial composition of the dung samples used.

Despite the differential effects of the OFs on the life history traits of both mosquito species tested, the effect on adult body size was consistent between the species. Another study found significant increases in *An. arabiensis* wing length when larvae were exposed to the cow dung derivative Fertilis [21]. The results of the present study add to this finding, and demonstrate that both *An. arabiensis* and *An. gambiae* s.s. develop into larger adults following larval exposure to complete cow dung and, to the best of our knowledge for the first time, following larval exposure to chicken dung. As increased body size is associated with greater fitness and survival in adults of *Anopheles* species [36, 65, 66], larval exposure to cow dung may result in increased adult fitness; however, the same cannot be said for chicken dung due to the high mortality and reduction in adult production associated with it.

Overall, the results of the present study suggest that, in comparison to *An. gambiae* s.s., developing *An. arabiensis* may be more tolerant of chicken dung at low levels in the aquatic environment. The survival of *An. arabiensis* at a chicken dung concentration of 0.25 g/100 ml was unaffected, and although adult production was reduced, it was not reduced to the same extent as in *An. gambiae* s.s. Therefore, when exposed to chicken dung, a larger number of *An. arabiensis* immatures may develop into adults, compared to *An. gambiae* s.s. The explanation for this relative tolerance of *An. arabiensis* is unclear, although it is known that discrete populations of this species have adapted to breed in organically polluted habitats [67], suggesting a degree of plasticity in their tolerance of organic pollutants.

The observations of this study may not reflect the effects of cow or chicken dung on mosquitoes in the field; however, the putative larvicidal qualities of chicken dung warrant further field-based research into its potential. Moreover, the application of chicken dung to aquatic habitats has been shown to benefit several families of invertebrate predators of mosquitoes [54], which have been demonstrated to effectively control larval abundances of *An. gambiae* s.l. [68], even in the presence of alternative prey [69]. In addition, chicken dung application in rice fields has been found to reduce rice crop damage by several pest insects via the promotion of terrestrial predators [70], suggesting that chicken dung may be used as a means of controlling both malaria vectors and pests of the rice plants themselves as a form of integrated vector/pest management.

It is uncertain how the application of cow and chicken dung in agricultural settings may affect the transmission of malaria, though many factors associated with mosquito life history traits also modify vectorial capacity. Larval survival is the key contributor to adult productivity, which in turn is the primary determinant of vector density [71], which is positively associated with biting rates [72]. Larger adults have increased survival, enhanced fecundity, and in the case of males, greater mating success, all of which may also result in increased vector densities, leading to increased vectorial capacity [36, 37, 65, 66, 73]. As exposure to cow dung had no impact on larval survival and resulted in larger adults, its application may lead to enhanced malaria transmission via increased vector densities, which are associated with higher biting and sporozoite rates [65]. Given that cow dung is a widely used organic fertiliser in African rice cultivation and its application is expected to increase to meet sustainable agriculture goals [20, 74], these results suggest that its use may enhance the fitness of vector populations. In contrast, although chicken dung also resulted in the production of larger adults, the substantial reduction in larval survival and adult production it induced would be of of greater importance and, therefore, may lead to a reduction in vector densities. Considering all of this, exposure to cow dung may lead to the enhancement, and chicken dung to the diminution, of a population’s vectorial capacity, though this requires substantiation in the field.

## Conclusions

The findings of this study show, to the best of our knowledge for the first time, the impact of chicken dung, a common OF, on the life history traits of *Anopheles gambiae* s.l. Cow and chicken dung had significant effects on the life history traits of the two dominant malaria vectors of Africa, *An. gambiae* s.s. and *An. arabiensis*. Overall, cow dung exposure may provide a fitness advantage to developing mosquitoes, whereas chicken dung is clearly detrimental to them. The use of inorganic fertilisers has been demonstrated on numerous occasions to promote populations of *Anopheles* species, with implications for increased malaria transmission [57, 75, 76], whilst the production of these fertilisers also contributes significantly to anthropogenic climate change [77]. Considering its toxicity to developing *Anopheles* species, the use of chicken dung in rice cultivation may present an eco-friendly and novel approach to mosquito control that may simultaneously reduce the malaria burden and satisfy the nutrient demands of growing rice; in contrast, the use of cow dung should be avoided. In the context of malaria transmission, the use of OFs in rice cultivation should be approached with caution, and their composition and dosage carefully considered. Further study should be directed towards investigating how OFs may affect *Anopheles* species populations in field-based settings.

## Data Availability

All the data have been presented in the manuscript and are available upon reasonable request.
